# miR-145-3p Hampers the Malignant Progression of Esophageal Carcinoma via CXCL5 Downregulation

**DOI:** 10.1155/2022/5418356

**Published:** 2022-07-29

**Authors:** Gang Chen, Zhihua Teng, Zhouyu Zhu, Xing Li

**Affiliations:** Department of Thoracic Surgery, The Second Affiliated Hospital Zhejiang University School of Medicine, Hangzhou Zhejiang Province, China 310000

## Abstract

Esophageal carcinoma (EC) is the most prevalent malignant tumor that occurs frequently worldwide. The early diagnostic biomarkers are crucial for EC treatment. miRNA can regulate EC progression, with diagnostic and prognostic value. Herein, differentially expressed miRNAs and mRNAs (DEmRNAs) in EC were predicted based on TCGA database. The target mRNAs of miRNA were predicted through databases, which were then intersected with DEmRNAs. Next, the correlation between miRNA and candidate mRNAs was analyzed. qRT-PCR was introduced to analyze expression of miR-145-3p and CXCL5 mRNA in EC cell lines, and western blot was performed to assess protein expression of CXCL5. Cell proliferation, migration, invasion, and apoptosis in EC were examined through CCK-8, wound healing, Transwell invasion, and flow cytometry assays. Moreover, targeting relationship between miR-145-3p and CXCL5 was verified through luciferase reporter gene analysis. The experimental results revealed a decreased miR-145-3p expression and an increased CXCL5 expression in EC. Enforced expression of miR-145-3p hindered proliferation, migration, invasion, and stimulated apoptosis of EC cells by repressing CXCL5. This study manifested that miR-145-3p may be a tumor suppressor in EC, and miR-145-3p/CXCL5 axis restrained the malignant progression of EC. These results supply an underlying target for prognosis and treatment of EC patients.

## 1. Introduction

Esophageal carcinoma (EC) is the most prevalent malignancy with the survival rate at 19% for all stages combined [1]. Despite significant advances in diagnostics, adjuvant, and neoadjuvant chemotherapy, the 5-year survival rate of EC patients remains below 30% [2]. About 50% of EC patients suffer metastases to distant lymph nodes or organs when they are diagnosed with this disease [3]. Metastasis remains the major fatal cause in EC patients. Hence, novel therapeutic regimens are required for EC treatment. It is essential to probe the pathogenesis of EC and seek out early diagnostic markers as well as novel treatment methods, thereby improving the survival of patients with EC. In this research, we aimed to explore the pathogenesis of EC and fig out novel biomarker.

MicroRNA (miRNA) is a type of small noncoding RNA, which can specifically recognize and bind to the downstream target mRNA 3′-untranslated region (3′-UTR), resulting in mRNA degeneration and translational suppression [4]. In studies on miRNAs relative to tumors, miR-145 is notably decreased in cancer tissues [5] and exerts vital regulatory functions in the development of multiple cancers [6, 7]. For instance, the miR-145-3p/HDAC4 axis hampers osteosarcoma cell proliferation and promotes autophagy and apoptosis [8]. miR-145-3p represses malignant behaviors of cervical cancer cells by targeting ZEB1 [9]. miR-145-3p restrains the growth of prostate cancer cells by targeting MTDH [10]. In fact, a previous study [11] had identified that upregulation of miR-145-3p could prominently repress ESCC progression. However, the downstream regulating mechanism and targets of miR-145-3p still need to be explored.

Chemokines are superfamily of signaling proteins or small cytokines secreted by cells, which can bind G protein-coupled receptors to target cells. CXCL5, as a subclass of CXC chemokines, acts as a ligand for CXCR2 [12]. A previous study has reported that the CXCL5/CXCR2 axis can facilitate tumor growth and angiogenesis and promote host cell infiltration and activation [13]. CXCL5 can also promote prostate cancer cell colony formation, proliferation, and migration [14]. CXCL5 facilitates proliferation and motility of NSCLC cells by activating PI3K/AKT and MAPK/ERK1/2 signaling pathways [15]. CXCL5 induces tumor angiogenesis in colorectal cancer by enhancing FOXD1 expression mediated by the AKT/NF-*κ*B pathway [16]. In addition, CXCL5 may be a potential prognostic indicator for many cancers, like prostate cancer [17], lung cancer [18], and colorectal cancer [19]. Nevertheless, little is known about the role of CXCL5 in esophageal cancer. Therefore, exploring the functional mechanism of CXCL5 in EC contributes to identifying novel diagnostic and prognostic markers of EC.

In this work, we aimed at explaining the role and modulatory mechanism of miR-145-3p in EC through bioinformatics analysis and cellular function experiments. These findings would contribute to improving our cognition of diagnostic and therapeutic strategies for EC.

## 2. Materials and Methods

### 2.1. Bioinformatics Methods

Expression profiles of mature miRNAs (normal: 13, tumor: 185) and mRNAs (normal: 11, tumor: 160) were acquired from The Cancer Genome Atlas (TCGA) database [20] (https://portal.gdc.cancer.gov/) (2020.05.25). Next, by using “edgeR” package, mRNA and miRNA expression in normal and tumor groups was subjected to differential analysis with |logFC| > 2 and padj < 0.05 as thresholds. After obtaining differentially expressed miRNAs (DEmiRNAs) and mRNAs (DEmRNAs), we identified the target miRNA of interest by literature review. Then, mRNAs sharing binding sites with the target miRNA were predicted through TargetScan (http://www.targetscan.org/vert_72/)(2020.05.25), mirDIP (http://ophid.utoronto.ca/mirdip/index_confirm.jsp) (2020.05.25), and miRWalk (http://mirwalk.umm.uni-heidelberg.de/)(2020.05.25) databases, which were then intersected with DEmRNAs. Finally, the target mRNA was identified through correlation analysis.

### 2.2. Cell Culture

Human normal esophageal epithelial cell line HEEC (BNCC337729) and EC cell lines EC109 (BNCC342591), EC9706 (BNCC339892), TE-1 (BNCC100151), and OE19 (BNCC338566) were accessed from BeNa Culture Collection (BNCC, China). EC9706 and TE-1 cells were incubated in RPMI-1640 medium (BNCC341471, BNCC, China), added with 10% fetal bovine serum (FBS, Gibco, USA). HEEC, EC109, and OE19 cells were prepared in DMEM (BNCC351841, BNCC, China) containing 10% FBS (Gibco, USA). All cells were cultivated at 37°C with 5% CO_2_ [21]. The cells were divided when the confluence reached 70–80%. We used PBS and 0.25% trypsin to rinsing and digest cells, respectively. Finally, the cell suspension was inoculated into new culture plates.

### 2.3. Cell Transfection

miR-145-3p mimics (miR-mimics) and its negative control (miR-NC), pcDNA3.1-CXCL5 plasmid encoding CXCL5 (oe-CXCL5) and its blank control pcDNA3.1 plasmid vector (oe-NC) were all synthesized by Sangon Biotech (China). With the Lipofectamine 2000 transfection reagent (Invitrogen, USA), EC cell line EC9706 was transfected with 20 nM oligonucleotides or 3 *μ*g of plasmids when cells have grown to 70% confluent [22]. miR-NC and oe-NC, miR-NC and oe-CXCL5, miR-mimics, and oe-CXCL5 were together transfected in EC9706 cell line. After 24 h, cells were gathered for following assays.

### 2.4. qRT-PCR

Total RNA was isolated with TRIzol reagent (Takara, Japan). Complementary DNAs (cDNAs) of miRNA and mRNA were synthesized with miRNA cDNA synthesis kit (Abm, Canada) and RT Master Mix (Abm, Canada), respectively. SYBR Green Master Mixs (SR1110; Thermo Fisher Scientific, USA) was utilized for PCR amplification. U6 was taken as the reference for miRNA while GAPDH was applied as a reference gene for CXCL5. Their relative expression was normalized through the 2^-*ΔΔ*Ct^ method [23]. The sequences of primers are displayed in Table 1.

### 2.5. Western Blot

Cells was lysed in radioimmunoprecipitation assay buffer (Thermo Fisher Scientific, USA) supplemented with phenylmethanesulfonyl fluoride. Through bicinchoninic acid protein assay kit (Beyotime, China), proteins were quantified. Afterwards, proteins underwent sodium dodecyl sulfate-polyacrylamide gel electrophoresis. Then, they were transferred to polyvinylidene fluoride membrane (Millipore, USA). Subsequently, 5% skimmed milk was recommended to block the membrane at room temperature. Next, the membrane was cultivated with primary antibody rabbit anti-CXCL5 (ab126763, 1 : 5000, Abcam, UK) or rabbit anti-GAPDH (ab181602, 1 : 10000, Abcam, UK) at 4°C overnight. After the membrane was washed, it was cultured with the secondary antibody goat anti-rabbit IgG (ab205718, 1 : 5000, Abcam, UK) under routine temperature for 2 h. Finally, the visualized protein signal was quantified with an enhanced chemiluminescence kit (GE Healthcare, USA), and analysis of band densities was completed using ImageJ software (National Institute of Health, USA). Relative CXCL5 protein expression was normalized to that of GAPDH [24].

### 2.6. CCK-8 Assay

CCK-8 assay was conducted as described previously to assess cell proliferation [22]. EC9706 cells were cultured in a 96-well plate (2 × 10^3^ cells/well) with RPMI-1640 medium (10% FBS). Then, cells were cultured for 0, 24, 48, 72, and 96 h, and 10 *μ*L CCK-8 reagent (CK04; Dojindo Laboratories, Japan) was supplemented into each well for another 2 h of incubation. Optical density value was assessed at 450 nm using a microplate reader (PerkinElmer, USA). This assay was conducted for 3 times.

### 2.7. Cell Invasion Assay

The 24-well plates and 8 mm aperture chambers (BD Biosciences, USA) were utilized in cell invasion assay. EC9706 cells transfected with miR-mimics for 24-48 h were collected and rinsed with phosphate-buffered saline (PBS), followed by digestion with trypsin (4 × 10^4^ cells/well). Next, cells were inoculated into the upper chamber (Corning, USA) that was precoated with Matrigel (BD Bioscience, USA) and added with serum-free RPMI-1640 medium. The lower chamber was supplemented with RPMI-1640 medium containing 10% FBS, followed by 24 h of cell incubation. Subsequently, uninvading cells in the upper chamber were removed, while 4% paraformaldehyde was recommended to fix the invading cells for 15 min, followed by 0.1% crystal violet for 20 min of cell staining. Finally, photos were taken using a BX43 light microscope (Olympus, Japan), and cell number was counted at 5 randomly selected fields in each well [25]. The assay was performed in triplicate.

### 2.8. Wound Healing Assay

This assay was performed as described previously [26]. A 6-well plate was recommended for incubation of EC9706 cells (3 × 10^5^ cells/well) at 37°C overnight. After 90% confluence of cells reached, a 20 *μ*L micropipette tip was applied to scrape cell monolayer to make wounds. The detached and damaged cells were carefully removed with PBS for 3 times, whereas the remained cells were incubated in a serum-free medium under routine temperature for 24 h. A Nikon Eclipse TE2000-U Inverted Microscope (Nikon, Japan) was utilized for observation and photograph of wounds at 0 and 24 h. The wound healing width was quantified and compared with baseline value. The assay was conducted in triplicate.

### 2.9. Dual-Luciferase Reporter Gene Assay

The assay was operated as described previously [27]. To identify the binding of miR-145-3p and the 3′-UTR of CXCL5, cells were cultivated in a 24-well plate at 60-80% confluence for 12 h. Next, the mutant (MUT) or wild-type (WT) 3′-UTR of CXCL5 that had putative binding sites of miR-145-3p was cloned into pGL3 vector (Promega, USA) to create CXCL5-MUT or CXCL5-WT luciferase reporter vector (pGL3-CXCL5-MUT/WT). Afterwards, 100 ng pGL3-CXCL5-WT/MUT and 40 nM miR-mimics or miR-NC were cotransfected into EC9706 cells. After 24 h, firefly luciferase activity was detected through dual-luciferase reporter gene assay (Promega, USA), and the result was normalized to Renilla luciferase. The assay was conducted in triplicate.

### 2.10. Cell Apoptosis Detection

After 48 h of incubation, cells were washed and resuspended in binding buffer, followed by 15 min of cell incubation with Annexin V-FITC (Nanjing KeyGen Biotech, China). Later, 5 *μ*L propidium iodide was supplemented to each sample, followed by 30 min of cell incubation in the dark at room temperature. The flow cytometry (FACSAria Cell Sorter, BD Biosciences, USA) was employed for cell apoptosis assessment within 1 h [28]. Three independent assays were performed.

### 2.11. Statistical Analysis

All measurement data were obtained from at least 3 independent experiments, which were dealt with GraphPad Prism 6.0 (GraphPad, USA), presenting as mean ± standard deviation. A *t*-test was applied for comparison between two groups.  ^∗^*p* < 0.05,  ^∗∗^*p* < 0.01, or ^∗∗∗^*p* < 0.001 was considered statistically significant.

## 3. Results

### 3.1. miR-145-3p Was Conspicuously Lowly Expressed in EC Cells

**Figure 1:**
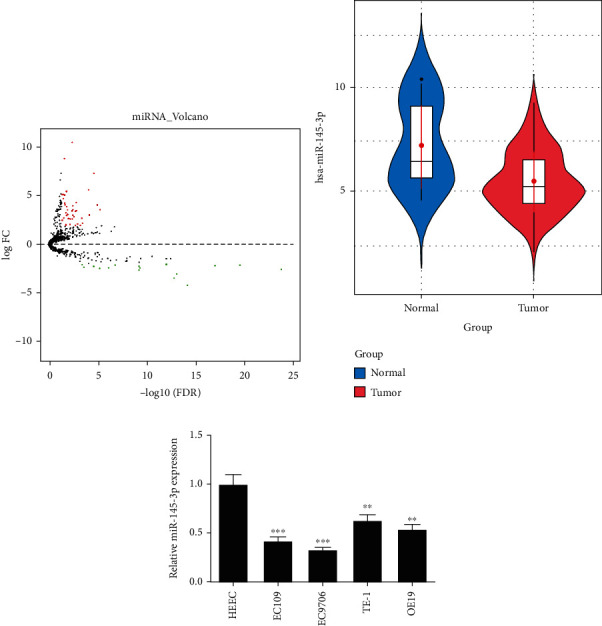
miR-145-3p is conspicuously lowly expressed in EC cells. (a) Volcano map of DEmiRNAs in normal and tumor groups in TCGA-ESCA dataset. Red indicates upregulated DEmiRNAs, and green indicates down-regulated DEmiRNAs. (b) The expression of miR-145-3p in normal samples (*n* = 13, blue) and tumor samples (*n* = 185, red). (c)The expression of miR-145-3p in EC cell lines (EC109, EC9706, TE-1, and OE19) and normal cell line HEEC. The difference comparisons are compared with the HEEC cell line. ^∗^*p* < 0.05,  ^∗∗^*p* < 0.01, and ^∗∗∗^*p* < 0.001.

As shown in Figure 1(a), 57 DEmiRNAs were obtained through differential analysis on miRNAs in TCGA-ESCA dataset, wherein miR-145-3p was prominently lowly expressed in EC tissues (*p* < 0.05) (Figure 1(b)). Meanwhile, several studies confirmed that miR-145-3p decreases in various cancers [8, 29, 30], and thus, miR-145-3p was selected as the research object. Afterwards, miR-145-3p expression in EC cell lines (EC109, EC9706, TE-1, and OE19) and normal cell line HEEC was detected by qRT-PCR, and we found that miR-145-3p was less expressed in EC cell lines (Figure 1(c)). Taken together, a decreased miR-145-3p expression could be observed in EC tissues and cells.

### 3.2. Overexpression of miR-145-3p Repressed Proliferation, Migration, Invasion, and Hastened Apoptosis of EC Cells

**Figure 2:**
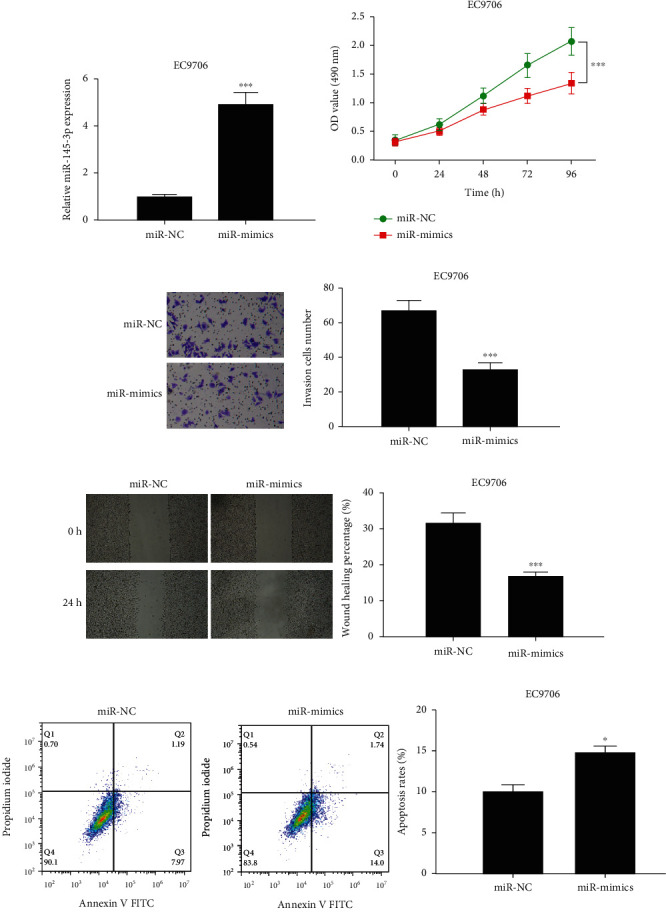
Overexpression of miR-145-3p represses malignant progression of EC cells. (a) Detection of the transfection efficiency of miR-145-3p in EC9706 cells. (b) The proliferative ability of EC9706 cells in miR-NC and miR-mimics groups. (c) The invasive ability of EC9706 cells in each transfection group (100×). (d) The migration of EC9706 cells in each transfection group (40×). (e) The cell apoptosis rate of EC9706 cells in each transfection group. All difference comparisons are compared with the miR-NC group.  ^∗^*p* < 0.05 and ^∗∗∗^*p* < 0.001.

The above assays revealed a prominent difference in miR-145-3p expression between EC cell line EC9706 and normal cell line. Therefore, EC cell line EC9706 was chosen for subsequent functional assays. First, overexpression of miR-145-3p was transfected into EC cell line EC9706 and was then subjected to qRT-PCR. As illustrated in Figure 2(a), the miR-145-3p level was notably upregulated after overexpression treatment (*p* < 0.001), indicating that the transfection efficiency reached the requirement for follow-up assays. Next, the proliferative, migratory, and invasive abilities of EC cells transfected with miR-mimics were examined via CCK-8, wound healing and Transwell assays. In Figures 2(b)–2(d), compared with the control group, miR-145-3p upregulation markedly hampered cell proliferation, invasion, and migration (all *p* < 0.001). Additionally, it was revealed by cell apoptosis assay that upregulating miR-145-3p remarkably fostered cell apoptosis of EC cells (*p* < 0.01) (Figure 2(e)). These findings demonstrated the tumor inhibitory effect of miR-145-3p on EC.

### 3.3. CXCL5 Was Highly Expressed in EC

**Figure 3:**
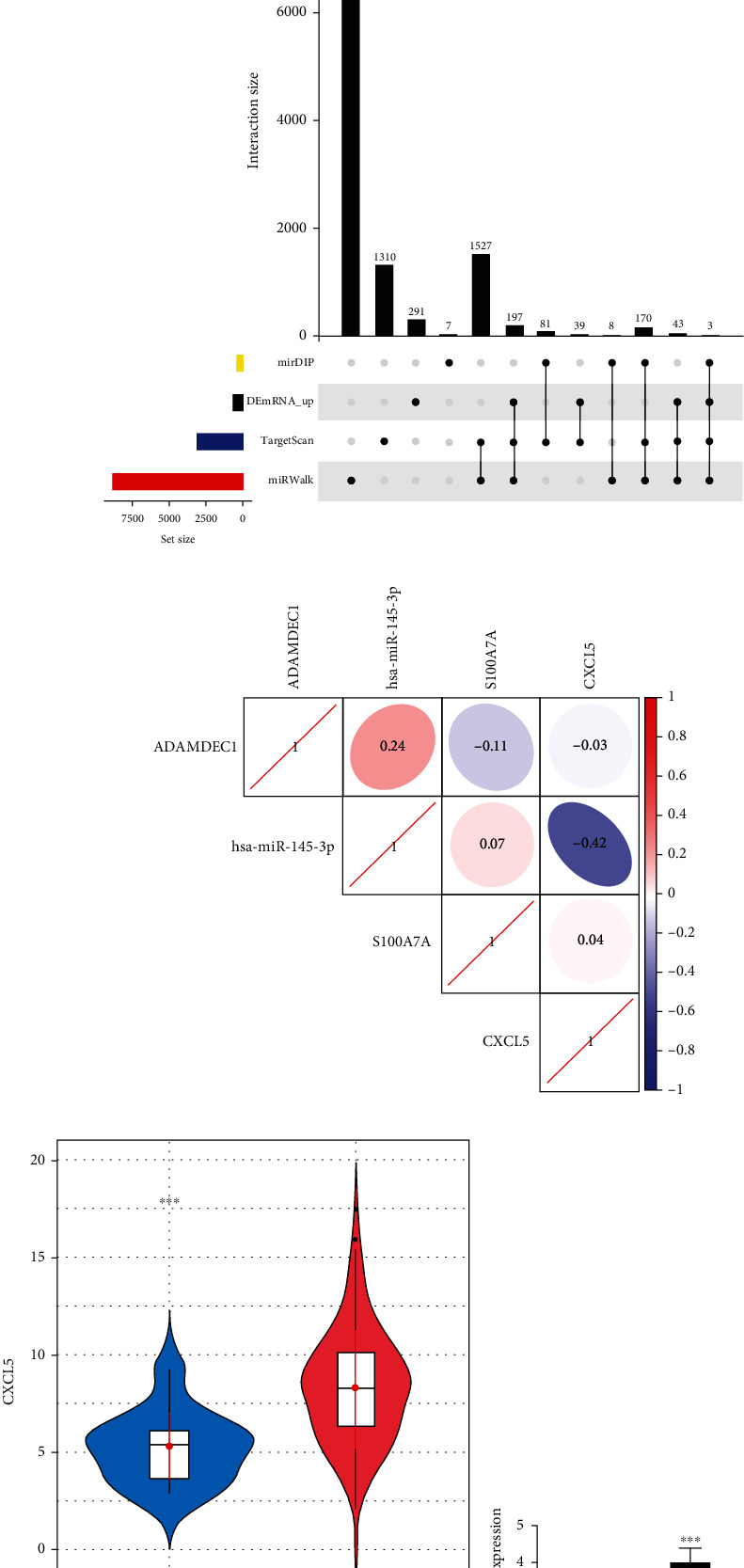
CXCL5 is highly expressed in EC cells. (a) Volcano map of DEmRNAs in normal (*n* = 11) and tumor (*n* = 160) groups in TCGA-ESCA dataset. (b) Upset map of the putative target mRNAs of miR-145-3p and upregulated DEmRNAs. Yellow indicates mirDIP database. Black indicates DEmRNAs in TCGA-ESCA dataset. Blue indicates TargetScan database. Red indicates miRWalk database. (c) Pearson's correlation analysis of gene candidates and miR-145-3p. (d) CXCL5 expression in TCGA database. (e, f) CXCL5 mRNA and protein levels in normal cells HEEC and EC cells (EC109, EC9706, TE-1, and OE19). The difference comparisons were compared with the HEEC cell line.  ^∗∗∗^*p* < 0.001.

To figure out the downstream regulatory mechanism of miR-145-3p in EC cells, a differential analysis was conducted on mRNAs in TCGA-ESCA dataset to screen out 1,227 DEmRNAs, containing 573 upregulated DEmRNAs and 654 downregulated DEmRNAs (Figure 3(a)). Next, target mRNAs of miR-145-3p were predicted through bioinformatics databases and were then intersected with the upregulated 573 mRNAs to obtain 3 DEmRNAs that had binding sites with miR-145-3p (Figure 3(b)). Pearson's correlation analysis was performed on miR-145-3p and these 3 DEmRNAs, in which CXCL5 had the highest inverse correlation coefficient with miR-145-3p (Figure 3(c)). Thus, CXCL5 was taken as the research object to further study its relationship with miR-145-3p. Data in TCGA-ESCA exhibited the increased expression of CXCL5 in EC tissues (*p* < 0.001) (Figure 3(d)). For assessment of CXCL5 expression in normal HEEC cells and EC cells (EC109, EC9706, TE-1, and OE19), qRT-PCR and western blot were applied. The CXCL5 mRNA level was upregulated in EC cell lines compared with the normal cell line (*p* < 0.001) (Figure 3(e)). Also, the CXCL5 protein level showed the same trend in EC cell lines (Figure 3(f)).

### 3.4. miR-145-3p Could Bind to CXCL5

**Figure 4:**
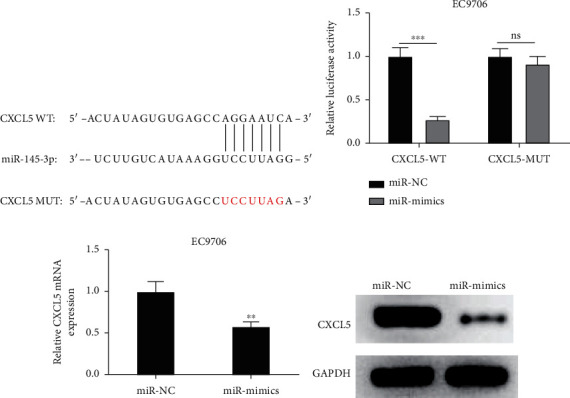
miR-145-3p downregulates CXCL5 expression. (a) The diagram of binding sequences of CXCL5-3′UTR-WT/MUT and miR-145-3p. (b) The luciferase activity of EC9706 cells in different treatment groups. (c, d) CXCL5 mRNA and protein expression in varying treatment groups. The difference comparisons are compared with the miR-NC group.  ^∗∗^*p* < 0.01 and ^∗∗∗^*p* < 0.001.

The binding sites of miR-145-3p and CXCL5 were obtained from TargetScan database. As illustrated in Figure 4(a), miR-145-3p had binding sites with CXCL5 3′-UTR. The result of Dual-luciferase reporter gene analysis showed that miR-145-3p mimics remarkably inhibited luciferase activity in the CXCL5-WT group (*p* < 0.001) (Figure 4(b)). The mRNA expression of CXCL5 in each treatment group of EC9706 cells was assessed through qRT-PCR, which was evidently downregulated when miR-145-3p was overexpressed (Figure 4(c)). The protein level of CXCL5 detected via western blot was also downregulated in EC9706 cells upon overexpressing miR-145-3p (Figure 4(d)). These findings displayed that CXCL5 was a target of miR-145-3p, and miR-145-3p could decrease CXCL5 expression in EC cells.

### 3.5. miR-145-3p Modulated Cell Proliferation, Migration, Invasion, and Apoptosis of EC through Targeting CXCL5

**Figure 5:**
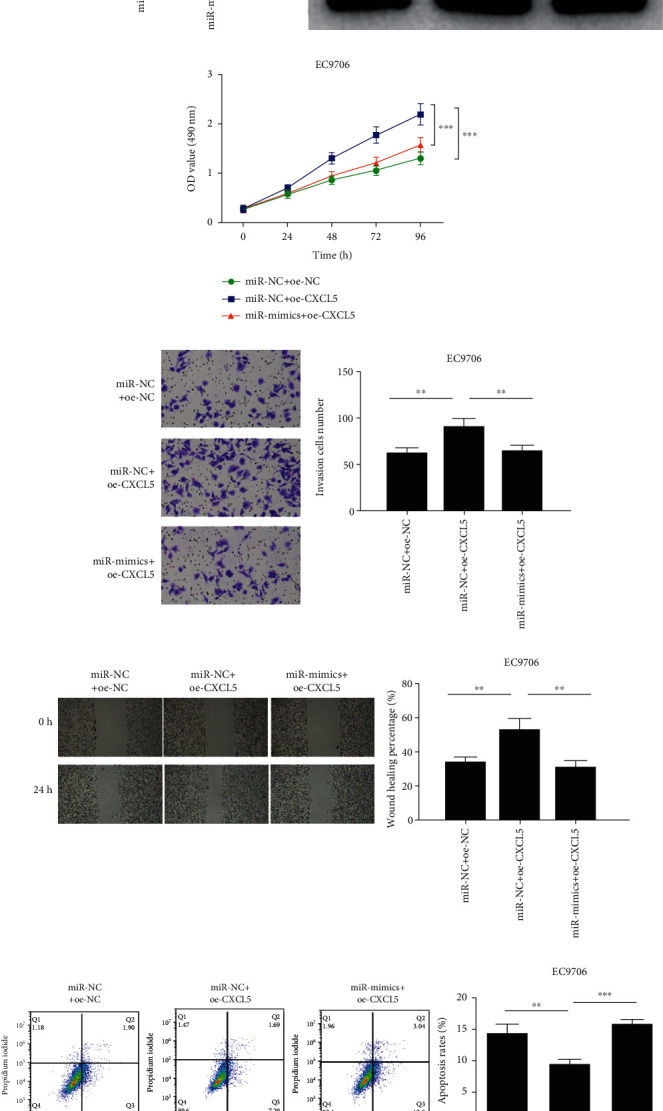
miR-145-3p modulates cell progression in EC through targeting CXCL5. (a, b) CXCL5 mRNA and protein expression in different treatment groups (miR-NC+oe-NC, miR-NC+oe-CXCL5, and miR-mimics+oe-CXCL5). (c) The proliferation of EC9706 cells in each treatment group. (d) The invasive ability of EC9706 cells in each treatment group (100x). (e) The migratory ability of EC9706 cells in each treatment group (40x). (f) The apoptosis rate of EC9706 cells in each treatment group. When the miR-NC+oe-NC group was compared with the miR-NC+oe-CXCL5 group, miR-NC+oe-NC was the control; when the miR-NC+oe-CXCL5 group was compared with the miR-mimics+oe-CXCL5 group, miR-mimics+oe-CXCL5 was the control. ^∗∗^*p* < 0.01 and^∗∗∗^*p* < 0.001.

miR-NC+oe-NC (control group), miR-NC+oe-CXCL5 (overexpression CXCL5 group), and miR-mimics+oe-CXCL5 (overexpression miR-145-3p and CXCL5 simultaneously) groups of EC9706 cells were constructed for investigation of the impact of miR-145-3p and CXCL5 on the malignant progression of EC cells. CXCL5 mRNA and protein levels in different transfection groups were assessed via qRT-PCR and western blot, respectively. As illustrated in Figures 5(a) and 5(b), CXCL5 expression was noticeably upregulated in the miR-NC+oe-CXCL5 group (*p* < 0.001), which was restored in the miR-mimics+oe-CXCL5 group (*p* < 0.001). Later, CCK-8, Transwell, and wound healing assays were carried out to measure the proliferative, invasive, and migratory abilities of EC cells (Figures 5(c)–5(e)). Compared with the miR-NC+oe-NC group, upregulation of CXCL5 fostered the proliferative, invasive, and migratory abilities of EC (*p* < 0.01). Besides, simultaneously transfecting miR-mimics could restore the promotion of overexpression of CXCL5 on the proliferative, invasive, and migratory abilities of EC cells (*p* < 0.01). Cell apoptosis rate was assessed via flow cytometry. As presented in Figure 5(f), overexpression of CXCL5 could inhibit EC cell apoptosis, while upregulation of miR-145-3p and CXCL5 at the same time could reverse the inhibition of CXCL5 on EC cell apoptosis (*p* < 0.001). In summary, our experiments showed that CXCL5 fostered cell function, and its promoting effects were reversed by miR-145-3p mimics in EC cells.

## 4. Discussion

More and more investigations illustrated that numerous miRNAs play imperative roles in biological processes including migration, invasion, transformation, and apoptosis. For example, miR-126 inhibits apoptosis and autophagy of esophageal squamous cell carcinoma (ESCC) [31]. miR-32 accelerates ESCC progression via regulating CXXC5/TGF-*β* pathway [6]. miR-203 suppresses invasion and migration of EC cells, and it can be used as a prognostic indicator for EC patients' outcomes [21]. The suppressing impact of miR-145-3p in varying cancers was reported. For instance, miR-145-3p represses cell proliferation and induces autophagy and apoptosis of osteosarcoma cells through targeting HDAC4 [8]. miR-145-3p or miR-145-5p hinders the proliferation and metastasis of prostate cancer cells through MTDH suppression [10]. This investigation manifested the low expression of miR-145-3p in EC. A series of functional experiments displayed that upregulating miR-145-3p hindered the malignant progression of EC, confirming that miR-145-3p functioned as a tumor inhibitor in EC.

At present, few studies are probing the regulatory mechanism of miR-145-3p in EC. CXCL5 may be one of downstream targets of miR-145-3p through bioinformatics methods. Thus, we studied the regulatory impact of miR-145-3p on CXCL5 expression in EC cells. Regarding regulatory role of CXCL5 in tumors, an existing study demonstrated that in colorectal cancer, CXCL5 fosters FOXD1 expression mediated through the AKT/NF-*κ*B pathway to induce angiogenesis [16]. Another study pointed out that apoptosis-mediated CXCL5 hastens inflammation and osseous metastasis of prostate cancer [17]. Nevertheless, the specific role and underlying mechanism of CXCL5 in EC are still unclear. Therefore, through cellular biological experiments, this study authenticated CXCL5 was highly expressed in EC cells. CXCL5 was directly targeted by miR-145-3p, and overexpression of CXCL5 could facilitate cell proliferation, invasion, and migration and repress cell apoptosis. Furthermore, rescue experiments manifested that overexpression of CXCL5 notably promoted malignant progression of EC cell lines, and its facilitation effects could be reversed when miR-145-3p was overexpressed. Together, these findings provided the potential molecular mechanism of CXCL5 in of EC cells, which lay the groundwork for future research into CXCL5 as a cancer modulator and help the generation of novel regimens for EC treatment.

In summary, this study confirmed that miR-145-3p is a key factor in suppressing cell proliferation, invasion, and migration. We also confirmed that CXCL5 acted as a direct target of miR-145-3p, which mediated antitumor effect of miR-145-3p in EC. This finding reveals the regulatory mechanism of miR-145-3p in EC progression to provide novel therapeutic targets for EC and deepens our understanding of the function of CXCL5 in tumor progression and its upstream regulatory mechanism, which provides new insight and a new entry point for targeted therapy of EC.

## Figures and Tables

**Table 1:** Primer sequences in qRT-PCR.

	Primer sequences	
miR-145-3p	Forward primer	5′-GCCCTGTAGTGTTTCCTACTT-3′
	Reverse primer	5′-GTGCAGGGTCCGAGGT-3′
U6	Forward primer	5′-CTCGCTTCGGCAGCACA-3′
	Reverse primer	5′-AACGCTTCACGAATTTGCGT-3′
CXCL5	Forward primer	5′-GCCTCCCTGAACGGGAAG-3′
	Reverse primer	5′-CAGTTTTCCTTGTTTCCACCGTCCA-3′
GAPDH	Forward primer	5′-GGACCTGACCTGCCGTCTAG-3′
	Reverse primer	5′-GTAGCCCAGGATGCCCTTGA-3′

## Data Availability

The data used to support the findings of this study are included within the article.
